# American Medical Association

**Published:** 1857-07

**Authors:** 


### ARTICLE XI.

### *American Medical Association.*

NASHVILLE, *May* 5, 1857.

WE are indebted to the courtesy of the editors of the Nashville Journal of Medicine and Surgery, for advanced sheets of the June number, from which we make the following abstract.
*Eds.*

The Association met at 11 o'clock, in the Representative Hall of the State Capitol, the president, Dr. Zina Pitcher, of Michigan, in the chair, and upon his right Dr. W. K. Bowling, of Tennessee, one of the vice-presidents. Dr. Wm. Brodie, of Michigan, and Dr. R. C. Foster, secretaries, were present.

The meeting having been duly organized, the first business in order was stated by the chair to be the reception of the report of the committee of arrangements.

Dr. C. K. Winston, chairman of the committee of arrangements, on behalf of the committee, and of the medical profession of the city generally, extended a sincere and cordial welcome to the members of the Association, in a few pertinent and appropriate remarks.

Dr. Winston then proposed that the roll of delegates, who had registered their names, should be read. The roll having been called, it appeared that twenty states were represented.

On motion of Dr. C. K. Winston, Drs. Felix Robertson, J. Shelby and J. Overton were made permanent members of the Association.

The number of delegates who had then registered their names was found to be one hundred and forty-six.

After the appointment of the nominating committee, the president delivered the annual address, whereupon, on motion of Dr. Flint, of Ky., the thanks of the Association were unanimously tendered to the president for his very able and interesting address, and that it be referred to the committee on publication.

The president informed the convention that Dr. F. Campbell Stewart, of N. Y., Dr. Alden March, of N. Y., Dr. Isador Gluck, of N. Y., and Dr. Pancoast, of Penn., had been appointed to represent this Association in foreign scientific bodies.

The committee on medical topography and epidemics being called, a communication from Dr. J. C. Watson, of Maine, was read, asking for further time to make a report, which was granted.

Dr. James Mauran, of the committee on medical topography and epidemics, for Rhode Island, being called for, the secretary read his apology, which was accepted. Dr. Peregrine Wroth, of same committee, for Maryland, sent in his report, with accompanying reports of Drs. A. M. White and Edmund E. Waters, which were received, and referred to the committee on publications. Dr. W. F. Sutton, of same committee, for Kentucky, sent an apology, and asked for further time, which was granted. The members of the same committee for the states of New Hampshire, Vermont, Massachusetts, New York, New Jersey, Pennsylvania, Delaware, Virginia, District of Columbia, South Carolina, North Carolina, Tennessee and Minnesota, being called, no reports were made.

The delegates from Connecticut and Louisiana being absent for the time, the consideration of their reports was postponed until to-morrow.

A report from Dr. J. F. Posey, of Georgia, was presented by Dr. Arnold, and subsequently withdrawn by him for the purpose of preparing an abstract of it.

The committee on nominations then appeared, and through their chairman, Dr. J. B. Lindsley, reported the following officers of the Association for the ensuing year, viz.

*President.*—Dr. Paul F. Eve, of Tennessee.

*Vice-presidents.*—R. J. Breckinridge, of Kentucky, D. M. Reese, of New York, W. H. Byford, of Indiana, and Henry F. Campbell, of Georgia.

And on motion of Dr. Arnold, of Georgia, the report was accepted.

*Second day.—Nashville, May* 6, 1857.

The Association met pursuant to adjournment. The minutes of yesterday were read and adopted.

The newly elected officers were then inducted into their respective seats.

Dr. Eve, of Tennessee, in taking the chair, addressed the Association in a few pertinent and able remarks.

Dr. Hooker, from the committee on medical topography and epidemics for the state of Connecticut, being called on for his report, arose and explained that it was his understanding that the committee were to have three years in which to make their report, and at the end of that time he would either be prepared, or ask the indulgence of the Association for further time.

The report of Dr. Posey, of Georgia, on the same subject, being called for, Dr. Arnold, of Georgia, read an abstract of the report of Dr. Posey, all of which, on motion of Dr. Palmer, of Michigan, was referred to the committee on publication, under a suspension of the rule.

On motion of Dr. Wood, of New York, the reports which were presented yesterday were also referred to the committee on publication, under a suspension of the rule.

The state of Ohio being called upon for a report upon its medical topography and epidemics, the secretary read an apology from Dr. G. Mendenhall, who asked further time in which to make a report, which was granted.

The states of Mississippi, Missouri, Michigan, Illinois, Indiana, Wisconsin, Iowa, California, and U. S. Navy being called, no response was made.

A telegraphic dispatch from Dr. J. M. Sims, of New York, was to report on the treatment of the results of obstructed labor, which was referred to the appropriate committee.

A communication was received from the Southern Methodist Publishing House, inviting the members of the Association to visit that establishment, which was received and accepted.

A communication was read by Dr. Lindsley, of Tennessee, from the Medical Association of Washington City, inviting the National Association to hold their next annual meeting in that city. On motion, the communication was referred to the committee on nominations.

A resolution was offered by Dr. Bartlett, of Wisconsin, tendering a vote of thanks to the late president of the Association, Zina Pitcher, for the able manner in which he has presided over the deliberations of this body, which was unanimously adopted.

The reports of special committees for 1856-'57 being next in order, they were called in order, as follows:

Inflammation—its Pathology, etc. Dr. E. R. Peaslee, Maine; asked further time. Referred.

Anatomy and Histology of the Cervix Uteri. Drs. H. Hutchinson and Charles E. Isaacs, New York; no report.

Treatment of Cholera. Dr. J. Taylor Bradford, Kentucky; no report.

Treatment best adapted to each variety of Cataract, etc.—Dr. Mark Stephenson, New York; further time asked. Referred.

Causes of the Impulse of the Heart, etc. Dr. J. W. Corson, New York; a communication received. Referred.

Causes of Infant Mortality, etc. Dr. D. Meredith Reese, New York; read an abstract of his report, which was referred to the committee on publication.

The vererable Dr. Shelby, of Tennessee, being present, was was invited to a seat on the stand. His appearance was warm-acknowledged.

Dr. Hobbs, of Ill., offered the following resolution:

*Resolved*, That a committee on essays (not including Prize Essay,) be appointed, to whom all essays prepared by members for publication by this Association shall be referred, which committee shall transfer to the committee on publication all essays they judge worth publishing. The said committee on essays to make a full report of their proceedings to the Association at its next annual session; provided, authors of rejected essays, being informed of said rejection by said committee, shall have the privilege of withdrawing their essays from the report of the committee to the Association.

On motion of Dr. Palmer, of Michigan, the resolution was indefinitely postponed.

The secretary read a protest signed by Drs. Richard Arnold, J. Gordon Harvard, Pike Brown, Geo. P. Padford, against admitting the delegates from Oglethorpe Medical College. After explanations and some discussion,

On motlon of Dr. Gunn, of Michigan, the protest was laid upon the table; and,

On motion of Dr. Palmer, the whole subject was referred to a committee of three to be appointed by the chair.

Dr. Brodie, of Michigan, moved, as an amendment, that no faculty member of a medical college be appointed upon the committee, which was accepted by the mover.

The chairman appointed as such committee, Drs. Wistar, of Pa., Bemis, of Ky., and Gibbs, of S. C.

Dr. Felix Robertson, the oldest physician in Tennessee, being in attendance, was invited to a seat on the stand. He was greeted with marked consideration by the Association.

The committee on nominations was convened to transact important business.

The calling of special committees was resumed:

Spontaneous Umbilical Hemorrhage, etc. Dr. J. Foster Jenkins, New York. Further time asked. Referred.

Use of Instruments in Obstetrical Practice. Dr. Henry Carpenter, Pennsylvania. No report.

Measures to be Adopted to Remedy the evils existing in the present mode of holding Coroner's Inquests. Dr. Alexander J. Semmes, D. C. Report presented, and the following resolution offered:

*Resolved*, That committees of three in each state, territory, and the District of Columbia, be appointed, and that said committee be, and they hereby are authorized, in the name of this Association, to memorialize their respective legislatures, to pass such laws as will best carry into effect the objects of the foregoing report.

The resolution was adopted and referred.

Treatment of the Results of Obstructed Labor.—Dr. J. Marion Sims, New York. Previously acted upon.

True Position and Value of Operative Surgery, etc.—Dr. J. B. Flint, Kentucky. Further time asked; granted.

Causes and Cure of Indigestion, etc.—Dr. G. Volney Dorsey, Ohio. No report.

Medical Jurisprudence of Insanity, etc.—Dr. C. B. Coventry, New York. Further time asked; granted.

Human, Animal, and Vegetable Parasites.—Dr. Joseph Leidy, Pennsylvania. No report.

Value of strict attention to Position in the treatment of Diseases of the Abdomen.—Dr. M. D. Darnall, Indiana. No report.

Milk Sickness.—Dr. George Sutton, Indiana. No report.

Blending and Conversion of the Types of Fever.—Dr. Clark G. Pease, Wisconsin. Communication sent, but not received. Postponed.

Best Substitutes of Cinchona, ect.—Dr. B. S. Woodworth, Indiana. No report.

Use of Cinchona in Malarious Diseases.—Dr. Franklin Hinkle, Penn. Report furnished. Referred to committee on publication.

Nervous System in Febrile Disease.—Dr. Henry F. Campbell, Georgia. Verbal abstract of report given.

Laws Governing the Absorption and Deposit of Bone.—Dr. John Neill, Penn. No report.

Intimate Effects of Certain Toxicological Agents in the Animal Tissues and Fluids.—Dr. John W. Green, New York. No report.

Medical Topography and Fauna of Washington Territory. Dr. George Suckley, U. S. Army. Report presented and referred.

Flora of Washington and Oregon Territories.—Dr. James Cooper, New Jersey. Report presented aud referred.

Intimate Structure and Pathology of the Kidney.—Dr. Charles E. Isaacs, New York. Further time granted.

Diseases Incidental to Emigrants, etc.—Dr. Israel Moses, New York. No report.

Etiology and Pathology of Epidemic Cholera. Dr. T. W. Gorbon, Ohio. Partial report presented and referred.

Excretions as an Index to the Changes going on in the System.—Dr. H. A. Johnson, Illinois. No report.

Remedial Effects of Chloroform.—Dr. D. D. Thompson, Kentucky. No report.

Best Means of Causing an Increase of the number of Essays, etc.—Committee: Drs. Leidy, Wood, and Meigs, Pa. No report. Committee continued.

Changes Produced in Composition and Properties of Milk, etc.—Dr. N. S. Davis, Illinois. Communication read and time granted.

Stomatitis Materna.—Dr. McGugin, Iowa. Further time granted.

An abstract of the report of Dr. Fenner, of La., upon the medical topography of that state, was then read and referred.

Dr. Dunglison, of Pa. offered the following resolution, which was unanimously adopted:

*Resolved*, That in the death of Dr. Grafton, of Miss., the American Medical Association has lost a talented and useful member, and society a benefactor.

Dr. Caspar Wistar, chairman of the committee upon the admission of the delegates from Oglethorpe Medical College, reported in favor of their admission, which report was adopted.

The Secretary read the following preamble and resolutions, which where unanimously adopted:

*Whereas*, It has pleased God to remove by death our fellow member, Robert M. Porter, and because of his devotion to the interests of the profession of medicine, and his steady support of the American Medical Association,

*Resolved*, That this Association learned with unfeigned sorrow of his decease; and that they have lost a firm and intelligent supporter, and society a benefactor and friend.

Dr. T. Bullard of Ind., offered the following:

*Resolved*, That in the death of Dr. John L. Mothersett, this Association has lost a talented and useful member, and society a benefactor.

The Secretary read a communication from the Connecticut Medical Society, asking that the time for holding the meetings of the Association in northern cities be changed to a later period in the year. Referred to the committee on nominations, with instructions to make a report. Adjourned.

*Third Day.—Nashville, May* 7, 1857.

The Association met pursuant to adjournment. The minutes of yesterday were read and adopted.

Dr. Hoyt, from the committee of arrangements, read the names of additional delegates to the Association, who had arrived since the meeting of the Association yesterday.

The Secretary read a communication from Dr. Clark G. Pease, of Wisconsin, which accompanied his report on "Blending and Conversion of the Types of Fever."

Dr. Hooker, of Conn. moved that the report be referred to the committee on voluntary contributions.

Dr. McKinley moved to amend by having a portion of the report read, which was lost.

And the motion recurring to refer the report, it was carried.

Dr. Curry, from the committee on voluntary contributions, submitted a report recommending the following papers for publication in the transactions of the Association, which was accepted:

1st. A new principle of diagnosis in dislocations of the shoulder joint: by L. A. Dugas, M. D., professor of surgery in the medical college of Ga., Augusta; accompanied by four photographic plates illustrating the principle.

2d. Medical statistics of Washington Territory. By George Suckley, M. D., U. S. A., embracing—1st. Geological divisions of the Territory; its geology, meteorology, fauna. 2. White population and its diseases. 3. Native population, diseases, medical practice; causes of their rapid disappearance. Concluding remarks.

3d. Medical Flora of Washington and Oregon Territories. By J. G. Cooper, M. D.

All of which is respectfully submitted.

R. O. CURREY, *Chairman.*
R. T. EVANS,
GEO. R. GRANT.

Dr. Lindsley, from the nominating committee, submitted the following report:

Committe on nominations beg leave to report:

*Secretaries.*—Robert C. Foster, of Tenn.; A. J. Semmes, of Washington City.

*Treasurer.*—Casper Wistar, of Philadelphia.

For the next place of meeting, Washington City.

*Standing Committees.*

Committee of Publication.—Francis G. Smith, of Philadelphia, chairman; Caspar Wistar, of Philadelphia; R. C. Foster, of Nashville; A. J. Semmes, of Washington City; Samuel L. Hollingsworth, of Philadelphia; Samuel Lewis, of Penn.; H. F. Askew, of Delaware.

Committee on Prize Essays.—Grafton Tyler, of Georgetown, D. C., chairman; J. C. Hall, of D. C.; J. F. May, of D. C.; Thomas Miller, of D. C.; A. J. Semmes, of D. C.; Joshua Riley, of D. C.; W. J. C. Duhamel.

Committee of Arrangements.—Harvey Lindsley, chairman; W. J. C. Duhamel, Cornelius Boyle, P. H. Coolidge, G. M. Dove, A. Y. P. Garnett, Wm. P. Johnston, of D. C.

Committee on Medical Education.—G. W. Norris, of Philadelphia, chairman; A. H. Luce, of Ill.; E. R. Henderson, of S. C.; G. R. Grant, of Tenn.; T. S. Powell, of Ga.

Committee on Medical Literature.—A. B. Palmer, of Detroit, chairman; A. F. Alexander, of Ala.; J. M. Mosgrove, of Ohio; P. Cassidy, of Penn.; S. Pollak, of Missouri.

Vacancies in Committee on Medical Topography and Epidemics.—T. B. Shuford to fill the vacancy caused by the death of Dr. Grafton, of Miss. C. W. Parsons to fill the vacancy caused by resignation of Joseph Mauran, of Rhode Iseland.

*Special Committees.*

Spontaneous Umbilical Hemorrhage of the newly born.—J. Foster Jenkins, of New York.

Influence of Marriages of Consanguinity upon Offspring.—Dr. Bemis of Ky.

Functions of different portions of the Cerebellum.—E. Andrews, of Ill.

Causes of the Impulse of the Heart and the agencies which influence it in health and disease.—J. W. Corson, of New York city.

Treatment of the results of Obstructed Labor.—J. Marion Sims, N. Y.

Treatment best adapted to each variety of cataract, with the method of operation, place of election, time, age, &c.—Mark Stephenson, of N. Y.

Human, Animal and Vegetable Parasites.—Joseph Leidy, of Philadelphia.

Best substitutes for Cinchona and its preparations in the treatment of Intermittent Fever, &c.—B. S. Woodward, of Ind.

Intimate structure and pathology of the Kidney.—Charles E. Isaacs, of N. Y.

Etiology and Pathology of Epidemic Cholera.—T. W. Gordon, Georgetown, Broome Co., Ohio.

Inflammation of Cervix Uteri.—Henry H. Miller, of Louisville, Ky.

On Milk Sickness.—D. W. H. Byford.

Best means of causing an increase of the number of Essays. Drs. Leidy, Wood, Meigs, of Pa.

Changes produced in composition and properties of Milk.—N. S. Davis, of Ill.

Stomatitis Materna.—D. C. McCugin, Iowa.

On Criminal Abortion, with a view to its general suppression. H. N. Storer, of Boston.

The committee recommend that the committees ordered by the adoption of the resolutions accompanying Dr. A. J. Semmes report be filled by the several State societies.

On motion of Dr. Brodie, amended so as to refer the same to the officers of several State societies; carried.

The committee also recommend the amendment of the third article of the Constitution, in relation to *meetings*, by inserting after the words "first Tuesday in May," the words, "*or the first Tuesday in June*," and also by inserting after the words "shall be determined" the words "*with the time of meeting*."

Special committee on the present state of science, as regards the pathology and therapeutics of the reproductive organs of the female.—D. Fordyce Barker, of New York.

On Moral Insanity.—D. Meredith Reese, M. D., N. York.

On Calculi and Diseases of the Urinary Organs, in Iowa, Minnesota and Nebraska.—Dr. J. C. Hughes, of Keokuk.

On the nature, tendency and general treatment of Syphilitic Bubos.—Moses Gunn, of Detroit.

On Medical Education.—(By Dr. Currey's resolution)—Jas. R. Wood, of New York; Geo. R. Grant, Memphis, Tenn.; John Watson, New York; C. B. Nottingham, Macon, Ga.; Rene La Roche, Philadelphia, Penn.

To fill a vacancy in the committee on Medical Topography and Epidemics.—Dr. J. L. Cabel, Charlottesville, Va.

Dr. March moved that the report of the nominating committee be taken up, and each subject to which it refers be considered separately, which motion prevailed. That portion relating to nominations was then adopted.

The place of the next annual meeting of the association being the next subject in order, after some discussion, on motion of Dr. March, the report of the committee was adopted.

Dr. Lindsley moved that, as Dr. Semmes, one of the newly elected secretaries, was absent, Dr. Brodie, of Michigan, be elected secretary *pro tem.*, which was carried,

Dr. Pitcher offered the following resolution, which was unanimously adopted:

*Resolved*, That a committee of three be appointed, of which the president of the association shall be chairman, to communicate with the surgeon general of the army, the chief of the medical bureau of the navy, and the secretary of the treasury of the United States, with a view to secure the concurrence of these departments of the federal government, so that its contributions to the medical topography, the vital statistics, and the sanatary police of the nation, may be made tributary to the labors of this association.

The chairman appointed as such committee, Drs. Z. Pitcher, of Michigan, and R. H. Coolidge, of Kansas.

Dr. Yandall offered the following resolution:

*Resolved,* That this association reaffirm the principles respecting the right of constituent bodies announced in a report contained in Vol. V, of its transactions in the following terms:

"The faculty of every chartered medical college shall have the privilege of sending two delegates to the association, *Provided,* that the said faculty contains not less than six professors, who give one course of instruction, annually, of not less than sixteen weeks, on anatomy, materia medica, theory and practice of surgery, midwifery, and chemistry; and also that the said faculty requires that its candidates for graduation, among other requisites, shall have attended two full courses of lectures, with an interval of not less than six months between them, one of which courses must have been in their institution.

Dr. Breckinridge in the Chair.

Dr. Buchanan proceeded to discuss the resolution, and at the close of his remarks moved to lay it on the table, which was subsequently withdrawn.

Dr. Boring offered the following resolutions in lieu, which he proceeded to discuss:

*Resolved,* That this association has not the power to control the subject of medical education.

*Resolved,* That the great objects of this association are the advancement of medical science, and the promotion of harmony in the profession.

*Resolved,* That the attempt, upon the part of this body, to regulate medical education having most signally failed in its object, and already introduced elements of discord, any further interference with this subject would not only be useless, but calculated to disturb and distract the deliberations of the association.

Dr. Currey offered the following resolutions in lieu of the whole subject:

*Whereas,* The subject of medical education has been committed at each annual session to standing committees, and various suggestions have been proposed which the association has adopted, and recommended to private instructors and to the medical colleges.

*Resolved,* That a committee of five be appointed by the committee on nominations, as a special committee, to be composed of members who are in no respect connected with any medical school, to devise *a system of medical instruction,* to be presented for the consideration of this association at its annual session in 1858.

*Resolved,* That the proposed system shall set forth a uniform basis upon which our medical institutions shall be organized, as well as have reference to the best mode of securing the preparatory medical instruction to the student; and that consequently the legitimate subjects to be embraced in said system will include primary medical schools—the number of professorships in medical colleges, the length and number of terms during the year, the requisite qualifications for graduation, and such other subjects of a general character as to give uniformity to our medical system, and preserve harmony and friendly intercourse in the ranks of the profession.

*Resolved,* That, upon the adoption of the proposed system by the association, all institutions which may conform to it shall be entitled to representation at the annual sessions of this association and none others.

The subject was further discussed by several members of the association.

The motion of Dr. Hooker being in order, the previous question was called and the resolutions of Dr. Currey were adopted.

The committee on voluntary contributions reported in favor of the publication, in the transactions of the association, of the following paper: "On the blending and conversion of types in fever: by C. S. Pease, M. D., of Wisconsin. The report was adopted.

Dr. Lindsley offered the following amendment to the Constitution, which was seconded by Dr. Gunn:—"In Art. II, omit the words 'medical colleges;' and also the words 'the faculty of every regularly constituted medical college, or chartered school of medicine, shall have the privilege of sending two delegates.'"

The amendment lies over until the next meeting of the association, under a rule of the organization.

On motion of Dr. Palmer, the resolutions reported at the last annual meeting of the association by the committee on plans of organization for State and County medical Societies, were taken up and adopted.

Several resolutions were adopted, tendering thanks to the profession and the citizens of Nashville, the authorities of the state and city, the reporters and publishers of the city press, and to Dr. Wm. Brodie.

*Resolved*, That the state and county societies throughout the Union be requested to recommend to their members to purchase the transactions of the American Medical Association, and that their officers act as agents for the same.

On motion of Dr. Gunn, of Michigan, the association recognised the presentation of a pamphlet by Henry Frazer Campbell, M. D., claiming "priority in the discovery and naming of the excito-secretory system of nerves."

On motion of Dr. Byford, the association then adjourned *sine die*.

